# The effect of levodopa–carbidopa intestinal gel infusion long-term therapy on motor complications in advanced Parkinson’s disease: a multicenter Romanian experience

**DOI:** 10.1007/s00702-015-1496-z

**Published:** 2015-12-23

**Authors:** O. Băjenaru, A. Ene, B. O. Popescu, J. A. Szász, M. Sabău, D. F. Mureşan, L. Perju-Dumbrava, C. D. Popescu, A. Constantinescu, I. Buraga, M. Simu

**Affiliations:** Department of Neurology, University of Medicine and Pharmacy “Carol Davila” Bucharest, University Emergency Hospital Bucharest, 169 Splaiul Independentei, Sector 5, Bucharest, Romania; Department of Neurology, University of Medicine and Pharmacy “Carol Davila” Bucharest, Colentina Hospital, 19-21 Stefan cel Mare Street, Sector 2, 020125 Bucharest, Romania; Department of Neurology, University of Medicine and Pharmacy Targu Mures, 38 Gh Marinescu Street, 540139 Targu Mures, Mures Romania; Department of Neurology, Emergency Clinical Hospital Oradea, 65 Gh Doja Street, 410169 Oradea, Bihor Romania; Department of Neurology, University of Medicine and Pharmacy “Iuliu Hatieganu” Cluj Napoca, Cluj County Hospital, 3-5 Clinicilor Street, 400006 Cluj-Napoca, Romania; Neurology Rehabilitation Department, University of Medicine and Pharmacy “Gr. T. Popa” Iasi, Clinical Rehabilitation Hospital, 14 Pantelimon Halipa Street, 700661 Iasi, Romania; Department of Neurology, University of Medicine and Pharmacy “Victor Babes,” Timisoara County Hospital, 10 Iosif Bulbuca Street, 300736 Timisoara, Timis Romania

**Keywords:** Parkinson’s disease, Motor complications, Levodopa–carbidopa intestinal gel (LCIG), Quality of life

## Abstract

Chronic treatment with oral levodopa is associated with an increased frequency of motor complications in the late stages of Parkinson’s disease (PD). Continuous administration of levodopa–carbidopa intestinal gel (LCIG—Duodopa^®^, Abbott Laboratories), which has been available in Romania since 2009, represents an option for treating patients with advanced PD. Our primary objective was to report changes in motor complications after initiation of LCIG therapy. The secondary objectives were as follows: to determine the impact of LCIG therapy on the daily levodopa dose variation before/and after LCIG, to collect patient self-assessments of quality of life (QoL), and to study the overall tolerability and safety of LCIG administration. A retrospective analysis (2009–2013) of LCIG therapy and the experience in nine neurology centers in Romania was performed. The impact of LCIG therapy was evaluated by analyzing changes in motor fluctuations, dyskinesia and the patients’ QoL after initiating therapy. The safety of LCIG therapy was estimated by noting agent-related adverse events (AEs) and medical device-related AEs. In the 113 patients included, we observed a significant improvement in PD symptoms after initiation of LCIG therapy. The “on” period increased, with a mean value of 6.14 h, and the dyskinesia period was reduced, with a mean value of 29.4 %. The quantified non-motor symptoms subsided. The patients exhibited significant improvements in QoL scores. There were few AEs and few cases of LCIG therapy discontinuation. LCIG is an important and available therapeutic option for managing patients with advanced PD.

## Introduction

Levodopa is currently the most effective agent for symptomatic treatment of PD, particularly when bradykinetic symptoms become intrusive with respect to a patient’s motor abilities. However, while the exact percentage is difficult to estimate (Ahlskog and Muenter 2001), somewhere between 50 and 90 % of patients with PD develop motor complications and dyskinesia within 5–10 years of levodopa treatment (Olanow et al. 2001). Dyskinesia, the most invalidating side effect of oral l-dopa therapy, becomes increasingly frequent with long-term treatment and advanced disease and is one of the greatest disadvantages of the oral levodopa treatment for Parkinson’s disease. As the therapeutic window becomes narrower, fine-tuning between the “off” time and dyskinesia becomes more difficult with the use of oral therapies partly because the gastric passage severely interferes with the process. Furthermore, motor fluctuations represent the other end of the problem, also highlighting the relatively short half-life of levodopa. Therefore, these side-effects of levodopa therapy are likely due to both the pulsatile dopaminergic substitution pharmacological characteristics of all available oral levodopa formulations (immediate or extended release) and the potential gastric barrier to its absorption. Continuous administration of LCIG through intestinal infusion represents a therapeutic option for advanced PD. Studies have demonstrated that the levodopa plasma concentration is less time variable with LCIG than with tablets (Nyholm et al. 2003). Data regarding its effects have been systematically collected in countries in which LCIG has been approved for use in routine clinical practice.

This therapeutic option for managing patients with advanced PD has been available in Romania since 2009 and has been used ever since in nine tertiary neurology centers. Other therapeutic options, such as apomorphine (Poewe and Wenning 2000; Drapier and Vérin 2006) and subthalamic nucleus deep brain stimulation (Krach et al. 2003; Tir et al. 2007), are applicable only to specific patient populations because of the particular inclusion criteria, which usually take into account age, degree of independence, disease stage, complications and co-morbidities (Morgante et al. 2007; Antonini and Tolosa 2009). In Romania, the availability of deep brain stimulation surgery is restricted to a single center that receives limited funding, and apomorphine was not available during the study period. Therefore, in Romania during the study period, LCIG therapy was the most optimal and readily available treatment for patients with advanced PD.

We aimed to establish the therapeutic benefit of this treatment during the first 5 years of treatment, as quantified by changes in motor skills and quality of life (QoL) scores. We collected data regarding all of the safety endpoints, including administration of medications, percutaneous endoscopic gastrojejunostomy (PEG/J) procedure and compliance.

## Methods

### Patient selection

Our study is an open, retrospective observation of the medical records of all of the patients who received LCIG (Duodopa^®^, Abbott Laboratories) continuous infusion therapy via percutaneous endoscopic gastrojejunostomy (PEG/J) by means of a device (CADD-legacy-Duodopa-pump, Smiths Medical, MN, USA) at nine neurology centers in Romania (three centers in Bucharest, two centers in Cluj and one center in each of Oradea, Targu Mures, Iasi and Timisoara) from 1 January 2009 until the 30 September 2013. The data collection was approved by the local ethics committee of each center. All of the patients underwent a naso-jejunal test to evaluate their response to continuous administration of LCIG, and they were considered to be good responders.

### Clinical data

The efficacy of this treatment for controlling motor symptoms was evaluated by analyzing the patients’ diaries, focusing on the daily length of the “off” period and the daily percentage of “on” time with dyskinesias. These data were collected for each patient during oral anti-parkinsonian therapy (with levodopa alone or combined with other oral therapies) to establish a baseline and several months after LCIG treatment initiation, during a follow-up visit performed between 3 and 6 months after treatment initiation.

The patients’ self-evaluation of their QoL before and after LCIG therapy was measured using the 10-point Visual Analog Scale (VAS).

Furthermore, quantification of non-motor symptom variation before and after LCIG therapy was performed using the information gathered by clinicians at the time that they evaluated the patients.

Additionally for each patient, we collected the total 24-h dose of levodopa administered before and during LCIG therapy.

The overall tolerability and safety of LCIG administration were evaluated based on the reports of agent-related AEs and medical device-related AEs collected from the patients’ medical files.

The levodopa equivalent daily dose (LEDD) was calculated using a formula recommended by the LCIG provider in Romania, as follows LEDD = levodopa dose + levodopa dose < extended release > ×0.75 + levodopa dose × 0.33 < if associated with entacapone > +pramipexole × 100 + ropinirole × 20 + rotigotine × 30 + rasagiline × 100 + amantadine).

### Statistical analysis

The changes in “off” time and the percentage of time with dyskinesias before and after LCIG initiation were compared using the Wilcoxon matched pairs signed rank test. The statistical significance level used 0.05.

The variables were described using standard statistical measures (number of observations, mean values, and minimum and maximum values) or frequency tables.

## Results

In our study, we included a total of 113 patients diagnosed with advanced primary Parkinson’s disease (Hoehn and Yahr stage ≥3). There were 31 patients in Bucharest, 14 patients in Cluj, 2 patients in Oradea, 31 patients in Targu Mures, 19 patients in Iasi and 13 patients in Timisoara. Females comprised 40 % of the patients and males 60 %. The mean age was 64 years. The mean duration from the time of clinical diagnosis of Parkinson’s disease until LCIG therapy initiation was 12 years. The mean duration of LCIG therapy was 2.1 years (1–5) (Table 1).

**Table 1:** General characteristics of patients

Number of patients	113
Males (number, percent)	68 (60 %)
Females (number, percent)	45 (40 %)
Age at initiation of therapy (median, range)	65 (26–79 years)
Disease duration until LCIG infusion (median, range)	12 (3–35 years)
Dyskinesia before and after LCIG continuous infusion therapy (mean, min–max)	
Mean daily percentage of dyskinesia before LCIG	36.31 % (0–75 %)
Mean daily percentage of dyskinesia after LCIG	6.89 % (0–50 %)
Reduction in daily dyskinesia percentage	29.41 %
Levodopa dose before and after LCIG continuous infusion therapy (mean)	
Daily Levodopa dose (mg) before LCIG	967.74 mg
Daily Levodopa dose (mg) after LCIG	1570.04 mg
Increase in daily levodopa dose (mg) after LCIG	602.29 mg
QoL assessment with the 10 points VAS before and after LCIG continuous therapy initiation (mean, min–max)	
Score before LCIG initiation	1.97 (0–6)
Score after LCIG initiation	6.8 (2–10)
Increase of the score after LCIG initiation	4.83
Reported adverse events (number, percent)	
Total number of patients that reported AEs	58 (51 %)
Patients that had incidental AEs	44 (38.93 %)
Patients that had LCIG infusion therapy-related AEs	15 (13.27 %)
Patients that had dopaminergic therapy-related AEs	7 (6.19 %)
Patients that had LCIG administration system-related AEs	2 (1.76 %)
Patients that had PEG/J procedure-related AEs	3 (2.65 %)
Patients that had compliance-related AEs	3 (2.65 %)

*LCIG* levodopa–carbidopa intestinal gel, *AEs* adverse events, *PEG/J* percutaneous endoscopic jejunostomy

**Fig. 1:**
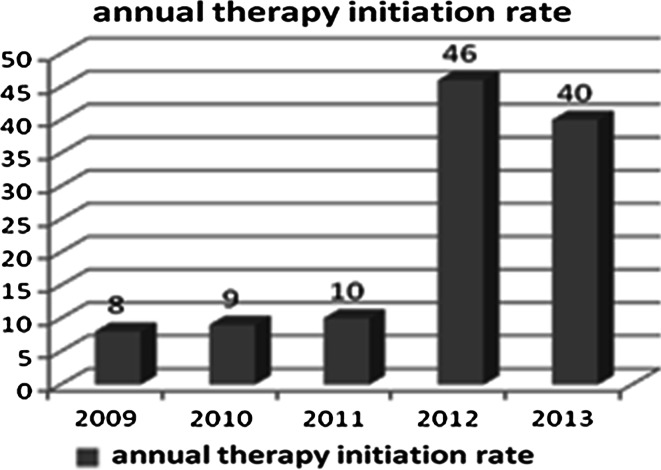
Annual therapy initiation rate

The increasing annual therapy initiation rate is shown in Fig. 1; the year 2013, which appears to have a lower initiation rate, was observed only until September. By the end of the observation period, the number of patients who still required LCIG treatment was 103.

### Benefit of LCIG therapy in terms of motor symptoms

The duration of the “off” time was significantly less after LCIG initiation (*p* < 0.0001); the comparison was made between the “off” period while on oral medication at the screening visit before LCIG therapy initiation and that after LCIG therapy initiation at a follow-up visit (3–6 months later). The mean “off” time before LCIG therapy was 7.5 h, whereas after LCIG therapy, it was 1.36 h. Thus, the mean reduction in the “off” period was 6.14 h.

**Fig. 2:**
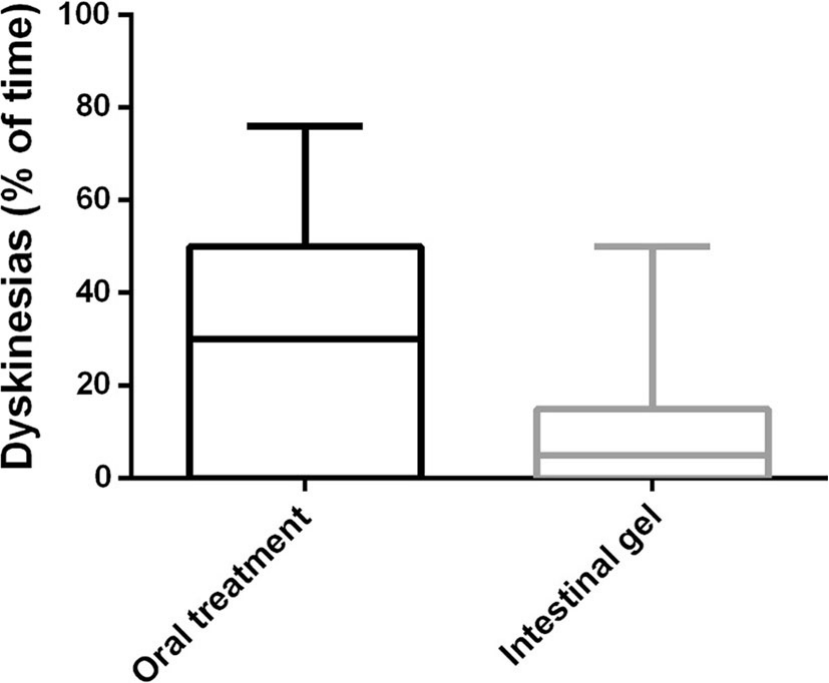
Percentage of daily dyskinesia before and after LCIG therapy

By providing continuous intrajejunal infusion, LCIG therapy facilitated better symptom control in our patients, significantly reducing the percentage of daily dyskinesia, with a mean of 29.4 % (from a mean of 36.3 % before LCIG to a mean of 6.9 % after LCIG) after initiation of LCIG therapy (*p* < 0.0001) (Table 1). Figure 2 shows the dyskinesia percentage before and after LCIG therapy.

The off-time in the male population (68 patients) was reduced with a mean of 7.03 h (range between 2 and 15 h) from the mean off-time before LCIG therapy of 8.36 h (range between 3 and 15 h). In the same subgroup, the mean percentage of dyskinesia before LCIG therapy was of 24.98 % (ranging between 0 and 75 %) and this improved after LCIG therapy with a mean of 17.24 % (ranging between 0 and 63 %). In the female population (45 patients) the off-time was reduced after LCIG therapy with a mean of 5.06 h (range between 0 and 9 h), from a mean of 6.08 h before LCIG therapy (range between 2 and 10 h). Improvement of dyskinesia daily percentage was with a mean of 33.2 % (range between 0 and 75 %) from the situation before LCIG therapy, when the dyskinesia mean percentage was of 43.44 % (range between 0 and 76 %) (Table 2).

**Table 2:** Reported non-motor symptoms

Non-motor symptom	Number of patients	Percentage	“on” period gain after LCIG
Sialorrhea	18	15.92	6.33
Taste disturbance	23	20.35	6.21
Nausea, vomiting	5	4.42	8.4
Constipation	44	38.93	6.7
Urinary incontinence	28	24.77	6.82
Weight loss	7	6.19	5.14
Hallucinations	6	5.3	6.41
Depression	56	49.5	6.43
Sexual dysfunction	11	9.73	6.9
Orthostatic hypotension	7	6.19	6.17
Excessive sleepiness	35	30.97	6.7
Insomnia	41	36.28	6.2
REM sleep disturbances	28	24.7	6.32
Restless legs syndrome	14	12.38	5.53
Excessive sweating	19	16.8	6.55
Impulse control disorders	6	5.13	7.33

Furthermore, in the group of patients aged 60 or younger, the mean reduction of off-time was of 5.7 h (range between 2 and 15 h), from a mean of 6.51 h spent in off-time (range between 2 and 15 h) before the LCIG therapy. They also had a mean reduction of 28.1 % in the daily dyskinesia percentage (range between 0 and 65 %) from the mean of 38 % of daily dyskinesias before LCIG therapy (range between 0 and 86 %).

**Fig. 3:**
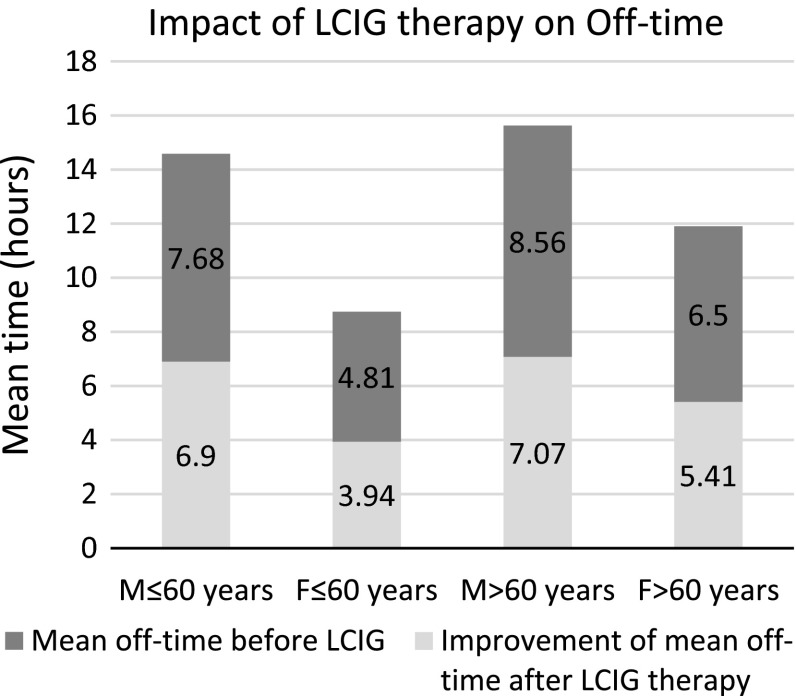
The impact of LCIG therapy on the daily off-time duration

In the group of patients above the age of 60, the mean reduction of the off-time was of 6.42 h (range between 0 and 13 h) from a mean of 7.75 h before LCIG therapy initiation (range between 2 and 13 h). The percentage of daily dyskinesia improved with a mean of 22.18 % (range between 0 and 75 %) from a mean of 30.30 % before LCIG therapy initiation (range between 0 and 75 %). Additionally, Fig. 3 depicts the impact LCIG therapy had on patients aged 60, or younger, vs. patients older than 60 years of age, respective of gender.

### Impact of LCIG therapy on the daily levodopa dose, before and after LCIG

LCIG continuous infusion therapy allowed an overall increase of the mean LEDD compared with the mean LEDD received while on oral therapy (from a mean of 967 mg/day to a mean of 1570 mg/day), with only 18 % of the patients exhibiting improved symptom control at lower levodopa doses compared with oral therapy (Table 1). The LEDD was calculated including all dopaminergic medication before LCIG therapy initiation.

Twenty patients (17.69 %) were administered an extended-release levodopa tablet after the LCIG had been stopped in the evening, and three patients (2.65 %) required 24-h continuous LCIG therapy because of poor motor and non-motor symptom control during the night, with only the conventional anti-parkinsonian therapies, essentially leading to severe impairment of sleep quality. Seven patients (6.19 %) received a dopaminergic agonist at bedtime (rotigotine for one patient, pramipexole for two patients, ropinirole for two patients, and a combination of ropinirole and rotigotine for one patient); nine patients (7.96 %) received an MAO inhibitor in the morning (rasagiline); and six patients (5.30 %) received amantadine for persistent dyskinesia.

### Patients’ self-assessments of QoL

A marked increase in their perception of their QoL was observed (Table 1), suggesting that LCIG therapy significantly improved the patients’ QoL (*p* < 0.01).

### Overall tolerability and safety of LCIG administration

**Fig. 4:**
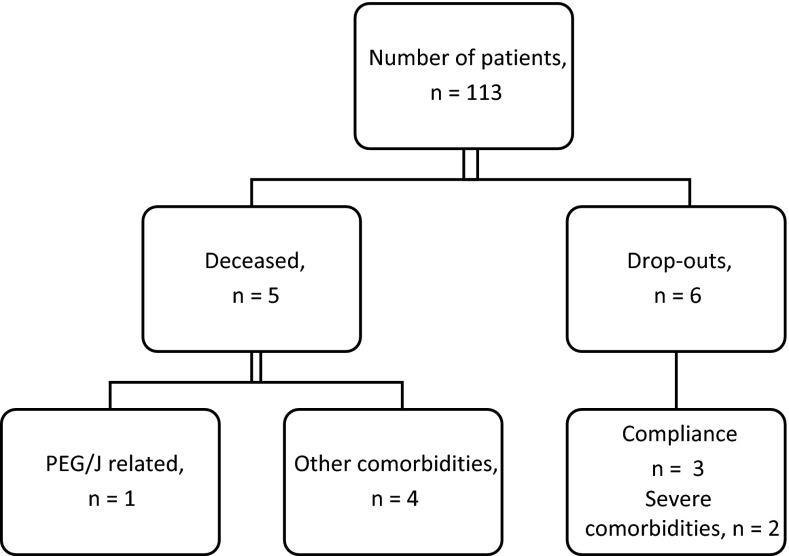
LCIG therapy cessation

A total of 58 (51 %) of 113 patients reported AEs. Of these AEs, 15 AEs (13.27 %) were related to LCIG infusion therapy, as follows: (1) seven AEs (6.19 %) were related to the medication itself (LCIG), meaning they were common adverse effects of the dopaminergic substitution therapy; (2) two AEs (1.76 %) were related to the LCIG administration system, meaning patients presented with problems of the intestinal tubes (calcification of intestinal tube—one case, detachment of intestinal tube—one case); (3) three AEs (2.65 %) were related to the PEG/J procedure, that is, complications secondary to the invasive placement of the intestinal tubes (post-interventional hiatal herniation—one case, abdominal wall leiomyoma—one case, sub-phrenic and hepatic abscess—one case). The rest of the reported AEs had no apparent relationship with the study treatment (Table 1). There were five deaths during the study period. One patient died as a result of the PEG/J procedure (pneumoperitoneum that resulted in peritonitis, septic shock and death), and the other four deaths occurred during the follow-up period (as a result of cardiovascular and respiratory co-morbidities). In seven patients, the treatment was ceased (one temporary and six definitive). The dropout reasons, in the six patients, were as follows: (1) compliance issues (lack of family support) in one patient; (2) severe dopaminergic adverse effects and cognitive decline in three patients (also posing compliance issues); and (3) digestive tract complications or other severe co-morbidities in two patients (Fig. 4).

Nine patients had newly diagnosed digestive tract pathologies during the study period. In one patient, the occurrence of a duodenal inflammatory reaction of unknown origin led to treatment cessation. The other diagnoses were as follows: superior digestive tract hemorrhage with melena and secondary anemia, sub-phrenic abscess, cascade stomach, hiatal herniation with intra-thoracic gastric volvulus, neoplasia of the hepatic angle of the colon, erosive esophagitis, erosive gastritis, and catarrhal cholecystitis with pancreatitis.

There were two patients with dopaminergic dysregulation syndrome (one case of punding three months after LCIG therapy initiation and one case of binge eating that was present before and after LCIG therapy initiation).

Three patients had significant weight loss (6 kg within 3 months from LCIG therapy initiation and 15 kg in the first year in one patient, 10 kg in the first month in the second patient, and 11 kg in 1 year in the third patient).

Axonal neuropathy was present in six patients (one had severe B_12_ deficiency, one had severe B_6_ deficiency, and one had diabetes mellitus (DM); in four patients, the neuropathy was hyperalgesic).

In three cases (2.65 %), LCIG therapy was not ceased during nighttime, because of severe sleep disturbances or severe motor symptoms during the night with oral levodopa supplementation. There were 20 patients (22.6 %) who were administered an extended-release levodopa dose after the LCIG therapy had been stopped; 7 patients received a dopaminergic agonist at bedtime; 9 patients received an MAO inhibitor in the morning; and 6 patients received amantadine for persistent dyskinesia. Of the 113 patients, 6 had psychiatric pathologies, 4 patients had associated dementia, and 6 patients had depression; all of these disorders appeared before LCIG therapy, and the patients received treatment for these conditions.

The impact of LCIG therapy on non-motor symptoms was also significant, with marked improvement in the majority of them. Unfortunately, there was an inconsistent screening for non-motor symptoms at the time of LCIG therapy initiation, making also the results of their follow-up less complete. However, of the symptoms that were noted, the majority exhibited definite improvement.

## Discussion

In Romania, LCIG therapy quickly became the most optimal and readily available option for treating patients with advanced PD. Our retrospective study is the first observation of the Romanian experience regarding patients who were diagnosed with advanced PD and who required advanced therapy, thus receiving LCIG continuous infusion therapy between 2009 and 30 September 2013.

Because additional patients were administered this therapy after the cutoff date, the results regarding the patient inclusion rate per year are only partial for the year 2013. Nevertheless, an increasing number of patients receive LCIG infusion therapy every year, thus suggesting that neurologists who work in outpatient clinics and in primary and secondary care hospitals feel an increased sense of confidence and have expertise in the use of LCIG therapy.

According to the majority of studies (Nyholm et al. 2012), the low dropout rate is likely due to the already proven efficacy of LCIG therapy. It has also been reported (Nyholm et al. 2012) that there might be a correlation between LCIG therapy duration and dropout incidence, thus suggesting an increased likelihood of dropping out of this treatment as the duration of the treatment increases. Our observation revealed the following reasons for dropout: very advanced PD with dopaminergic adverse effects and cognitive decline; patients becoming bedridden due to non-related pathologies, thus making LCIG treatment redundant; compliance issues; LCIG therapy-related complications. Despite these exceptions, LCIG infusion therapy resulted in homogeneous improvements in motor symptom control (with “off” period reduction and dyskinesia daily percentage reduction) and QoL, as reported by the patients. The observed complications precipitated by the procedure and pump system were, for the most part, due to anatomical and physiological particularities of the patients (e.g., cascade stomach, hiatal herniation, esophagitis, gastritis, and peptic ulcer); in one case, we found a non-specific inflammatory reaction of the duodenum that was resolved after tube removal.

The recognition of non-motor PD symptoms was non-homogeneous throughout the nine neurology centers in Romania, likely because of a lack of a definite consensus in this respect (Todorova et al. 2014), thus making it impossible to accurately determine the impact of LCIG on non-motor symptoms. Although motor symptoms have an important influence on how the patient perceives his or her QoL, non-motor symptoms are compelling runners-up, sometimes being more important than the cardinal symptoms of PD in determining how the patient’s QoL is perceived (Todorova et al. 2014). Given the constant improvement in QoL, as demonstrated by the 10-point VAS, it is safe to assume that levodopa-responsive non-motor symptoms were also improved by LCIG therapy (Antonini et al. 2015; Todorova and Ray Chaudhuri 2013). However, there is a need for additional research to define a consensus for non-motor PD symptom recognition and treatment (Todorova et al. 2014; Rascol et al. 2011; Antonini and Albin 2013).

Several studies have demonstrated a lack of difference in the daily levodopa dose with LCIG and oral therapies (Nyholm et al. 2005; Defer 2000), and other studies have demonstrated a decrease in the daily levodopa dose after LCIG (Nyholm et al. 2008). However, similar to a more recent report (Devos 2009), our observation demonstrated an increase in the mean daily levodopa intake. We also noted a consequent decrease in the mean daily dyskinesia percentage. This finding might indicate that patients tolerate higher daily doses of levodopa with obvious subsequent benefits (“off” period reduction and better QoL) without having to pay the price of pulsatile levodopa administration (oral therapy), which results in alternately high and low levodopa concentration profiles, thus leading to motor fluctuations (e.g., dyskinesia) (Devos 2009). The small number of patients that required night pumps, night oral extended-release levodopa tablets or adjuvant symptomatic agents (agonists or MAO inhibitors) further highlights, as other studies have demonstrated (Nyholm et al. 2012), that LCIG is efficient.

In our group of patients, adverse events related to LCIG therapy were rare. Indeed, the total number of AEs related to the mode of administration of LCIG therapy (including peri-procedural and immediate post-procedural complications of PEG/J placement, late complications of PEG/J and/or infusion system complications) tend to decrease over time. To our knowledge, this pattern is a result of the decrease in the first two categories mentioned, which are also the most common, considering that the adverse events profile in PD patients resembles that of the PEG/J tube in general (Fernandez et al. 2013). However, as other studies (Nyholm 2012; Devos 2009; Zibetti et al. 2013a; Nyholm et al. 2012; Honig et al. 2009) reported, adverse events related strictly to the infusion system (or the late complications of PEG/J) are present in a significant number of patients. Since in our group of patients an increasing number of technical problems was observed even after the cutoff date, we assume that an observation for a longer period of time may have yielded slightly different results, with respect to the number of LCIG therapy-related AEs. Moreover, patients with more advanced stages of PD, complicated with dementia, may be more prone to accidental tube displacement. Besides, drug-related complications, such as weight loss and axonal neuropathy, may lead to a variable degree of motor impairment, which could have the same consequences (Devos 2009; Antonini et al. 2013; Mancini et al. 2014). Nevertheless, considering the large number of patients who are currently receiving this treatment worldwide, there is room for improving the technical aspects of LCIG therapy and for minimizing device-related complications.

Overall, LCIG therapy was definitely beneficial in terms of controlling motor symptoms in our patients. Our observation is congruent with published data from other countries, thus confirming that LCIG therapy is an important tool for treating patients with advanced Parkinson’s disease.

The limitations of our study firstly lie in the shadow of the study design, as retrospective data collection sometimes revealed missing information. Secondly, since there was no initially established consensus on non-motor symptoms recording, data might be less revealing than we expected; we also observed a high variability between centers, in this respect. Furthermore, the relatively short observation time period could be seen as another limitation, as a longer follow-up would have probably revealed slightly different results.

## Conclusions

Our study demonstrated a homogeneous beneficial impact of LCIG therapy in treating patients with advanced PD, improving motor symptoms as well as their overall QoL, as reported by the patients and their families. Because our study was a retrospective observation, the impact of LCIG therapy on non-motor symptoms was not homogeneously recorded, and no statistical analysis could be performed. A challenging task for future research is to demonstrate the safety and the cost/benefit ratio of LCIG therapy in the long term.
